# The Role of the Mesopancreas in Periampullary Malignancies

**DOI:** 10.3390/cancers18091434

**Published:** 2026-04-30

**Authors:** Stephan O. David, Andrea Alexander, Lena Haeberle-Graser, Aslihan Yavas, Falko Rug, Ahmad B. Sultani, Sascha Vaghiri, Irene Esposito, Sami A. Safi, Wolfram T. Knoefel

**Affiliations:** 1 Department of Surgery (A), University Hospital Duesseldorf, Heinrich-Heine-University, Moorenstr. 5, 40225 Duesseldorf, Germany; andrea.alexander@med.uni-duesseldorf.de (A.A.); f.rug@hhu.de (F.R.); ahmadbaktash.sultani@med.uni-duesseldorf.de (A.B.S.); sascha.vaghiri@med.uni-duesseldorf.de (S.V.); sami-alexander.safi@med.uni-duesseldorf.de (S.A.S.); 2 Institute of Pathology, University Hospital Duesseldorf, Heinrich-Heine-University, Moorenstr. 5, 40225 Duesseldorf, Germany; lenajulia.haeberle@med.uni-duesseldorf.de (L.H.-G.); aslihan.yavas@med.uni-duesseldorf.de (A.Y.); irene.esposito@med.uni-duesseldorf.de (I.E.)

**Keywords:** distal cholangiocarcinoma, ampullary carcinoma, mesopancreatic excision, CRM

## Abstract

Background: Surgery and the perioperative management for periampullary carcinomas are translated from the more frequent ductal adenocarcinoma of the pancreatic head (hPDAC). After implementation of the pathological circumferential resection margin (CRM), true margin negativity dropped dramatically for hPDAC patients. The frequent infiltration of the mesopancreas (MP) is a causative factor for incomplete resection. It remains unknown if the oncological relevance of the MP remains exclusive for the hPDAC or if it can be translated into the operative management for periampullary carcinomas as well. Material and methods: Patients who received oncological pancreatoduodenectomies (PD) for dCCAs and ACs from 2015 to 2025 at our department were included in this study (n =100). The MP status was retrieved from the histopathological reporting. Results: MP infiltration was evident in 36.4% and 62.2% of the AC and dCCA patients respectively (*p* = 0.015). Across both tumour entities, mesopancreatic involvement emerged as a marker of significantly worse overall survival (AC: *p* = 0.002; dCCA: *p* = 0.013). Conclusion: Distal cholangiocarcinomas presented with a frequent infiltration into the mesopancreas. A positive infiltration status of the MP significantly correlated with incomplete resection status in ampullary carcinoma. In addition, MP infiltration proved to be an adverse prognostic factor for overall survival in periampullary carcinoma patients, underscoring its potential role in perioperative staging and its possible relevance for surgical decision-making. This is the first study revealing insights into the infiltrative prevalence of the MP in periampullary carcinomas. Mesopancreatic involvement may not be exclusive to pancreatic cancer and warrants further investigation in other periampullary malignancies.

## Simple Summary

The mesopancreas is defined as the retropancreatic tissue behind the pancreatic head extending towards the superior mesenteric artery and containing lymphatic, neural, and vascular structures. In pancreatic head cancer, the mesopancreas has gained increasing attention as a key anatomical and oncological compartment, as tumour spread into this region is associated with incomplete resection and worse outcomes. Consequently, surgical concepts targeting this area are becoming more widely established. In contrast, the role of the mesopancreas in other periampullary malignancies, such as ampullary carcinoma and distal cholangiocarcinoma, remains largely unexplored. This study aimed to investigate whether tumour infiltration into the mesopancreas also occurs in these entities and whether it has clinical relevance. By analyzing surgical and histopathological data from 100 patients, we demonstrate that mesopancreatic involvement is common, particularly in distal cholangiocarcinoma, and is associated with more aggressive tumour characteristics and reduced survival. These findings suggest that the mesopancreas may represent a relevant anatomical compartment in surgical treatment and risk stratification beyond pancreatic cancer, although its clinical implications require further investigation.

## 1. Introduction

Distal cholangiocarcinomas (dCCA) and ampullary carcinomas (AC) beside hPDAC are the most common periampullary carcinomas. ACs and dCCAs account for 0.2% and 3% of gastrointestinal cancers respectively, which underlines their low incidence [1,2]. Periampullary carcinomas and the ductal adenocarcinoma of the pancreatic head (hPDAC) share the same surgical approach, namely pancreatoduodenectomy (PD), in order to secure margin clearance and chances for long-term survival. Although these malignancies are considered to have an aggressive tumour biology, margin control significantly improves survival outcome underlining the importance of radical surgery [3,4]. Since PDAC is the cause for nearly 80% of all oncological pancreatic resections, most of the literature on perioperative management and oncological risk stratification arises from PDAC patients.

The implementation of the circumferential resection margin (CRM+/−) in 2004 according to the recommendations of the Royal College of Pathologists has revolutionized the histological assessment of pancreatoduodenectomy specimens. Using this approach, the anterior, posterior and medial surface of the pancreatic head are inked using different colours, subsequently, the specimen is sliced in an axial fashion and, finally, all three CRM components are assessed separately on a microscopical level [5,6]. The implementation of the so-called “1 mm rule” for true margin negativity (R0CRM−) has led to a significant reduction in true R0 resections which explains known high local recurrence rates for all three malignancies [7,8,9,10].

Particularly the medial and dorsal resection margins have been identified as key areas prone to tumour involvement [7,11]. While the ventral margin is of lesser oncological relevance, the dorsal margin in particular encompasses the mesopancreas. The mesopancreas refers to the tissue located circumferentially around the pancreas consisting of vessels, lymphatic, nerve and fatty tissue [12,13,14]. Due to the secondary retroperitoneal nature of the pancreas, the Treitz fascia and the Fredet fascia serve as landmarks for the ventral and dorsal borders of the mesopancreatic borders [12,13,15,16]. The idea is derived from similar concepts regarding complete mesocolic and mesorectal excision [17,18]. The dorso-medial aspects of the mesopancreas are located around the superior mesenteric artery; the site of embryologic rotation until the pancreas remains in its secondary retroperitoneal position [12,13]. Since its first surgical description in 2007, its clinical relevance has remained poorly investigated [19]. Studies by us and others [20,21,22,23,24] have revealed a surprisingly high infiltration rate of the mesopancreas, occurring in over 80% of PDAC patients. Furthermore, a standardized mesopancreatic excision has been shown to increase true R0 resection rates, enhance lymph-node yield, and reduce locoregional recurrence [20,24,25,26] but also to exhibit a favourable safety profile [26].

Building on these insights, it remains to be clarified whether mesopancreatic involvement also occurs in ampullary carcinoma and distal cholangiocarcinoma, and whether a risk stratification based on the degree of mesopancreatic infiltration and, subsequently, the consideration of mesopancreatic excision may likewise be justified in these tumour entities. Our study therefore aimed to generate initial evidence on the presence and potential clinical relevance of mesopancreatic infiltration in ACs and dCCAs. We hypothesize that mesopancreatic excision, which has already been standardized and routinely applied in selected high-volume centres for pancreatic ductal adenocarcinoma [11,14,24,25], could also play a pivotal role in securing local tumour control in periampullary malignancies.

## 2. Materials and Methods

**Figure 1:**
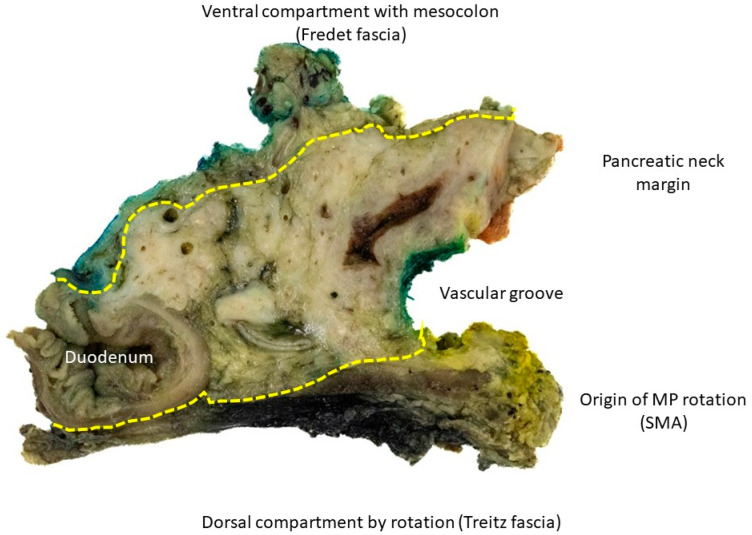
Representative surgical specimen demonstrated using the axial slicing technique. Key surgical landmarks are annotated to support standardized assessment. The dorsal boundary is demarcated by the Treitz fascia, and the ventral aspect by the Fredet fascia. The vascular groove indicates the course of the superior mesenteric artery (SMA). The dorsal medial pedicle of the mesopancreas represents the origin of mesopancreatic rotation.

All patients who underwent structured pancreatoduodenectomy (PD) for dCCAs and ACs with curative intent, irrespective of tumour stage and microscopic resection margin, at the University Hospital of Duesseldorf between 2015 and 2025 were enrolled in this study from a prospectively maintained database. The principles of mesopancreatic excision have already been discussed by us and others [1,2,3,27,28] and follow similar anatomical principles from colorectal surgery. Caused by the secondary retroperitoneal nature of the pancreatic head, the Treitz and Fredet fascia serve as anatomical landmarks and borders of the peripancreatic compartment (Figure 1).

Inclusion criteria are surgically resected non-metastasized dCCAs and ACs. All included patients have received upfront surgery. The staging system was updated to the 8th Edition of the UICC TNM classification of malignant tumours [4]. CRM analysis during macroscopical cross sectioning and histopathological evaluation according to LEEPP and the RCP was implemented in August 2015. The mesopancreatic infiltration status was taken from the histopathological reports. Cases in which the status was not initially provided in the pathological reporting were revisited by a pathologist experienced in the hepatopancreaobiliary field (L.H.-G.; I.E.).

For dCCA and AC patients, we additionally evaluated the infiltration status of the pancreatic tissue and duodenum during histopathological staging. For all included patients, the “1 mm rule” was implemented: a minimum margin clearance of 1 mm defined R0(CRM-), whereas margin clearances between 0 and 1 mm were judged as R0(CRM+).

### Statistics

The Wilcoxon test was used to analyze the differences in clinico-pathological data. The Mann–Whitney U test was used to examine numerical data and to correlate between clinico-pathological variables. For categorical data, the Chi-square test or Fisher’s exact test was applied. Survival probabilities were estimated by Kaplan–Meier analysis and compared with the log-rank test. Independent prognostic factors were identified by Cox proportional hazards regression. A forward stepwise multivariate Cox model was used for ampullary carcinoma (AC), while a bivariate Cox model including N-status and MP-status was computed for distal cholangiocarcinoma (dCCA). Analyses were performed using SPSS statistics for Windows (version 27.0; SPSS, Inc., Chicago, IL, USA). Statistical significance was defined as *p* < 0.05. The study was carried out in accordance with the guidelines of Good Clinical Practice and the Declaration of Helsinki. The study was approved by the Institutional Review Board (IRB) of the Medical Faculty, Heinrich Heine University Duesseldorf (IRB-no. 2020-1990).

## 3. Results

### 3.1. Demographic Data

In this study, a total of 100 patients resected with curative intent were included, comprising 55 patients with ampullary carcinoma (AC) and 45 patients with distal cholangiocarcinoma (dCCA). The median age at surgery was 66 years (31–83) for AC patients and 68 years (40–84) for dCCA patients. Among both entities, dCCA occurred predominantly in male patients (80.0%) compared with AC (56.4%; *p* = 0.018). Furthermore, dCCA patients tended to present at a more advanced T stage than those in AC (*p* = 0.003), and similarly, perineural invasion was significantly more frequent in dCCA (66.7%) compared with AC (27.3%; *p* < 0.001).

An R0CRM− resection was achieved more often in AC patients (89.1%) than in dCCA (71.1%; *p* = 0.039). Apart from these differences, the clinicopathological characteristics were largely comparable between the two groups. Further demographic data are summarized in Table 1.

### 3.2. Mesopancreas and Ampullary Carcinomas

Histopathological data were correlated with the infiltration status of the mesopancreas in AC patients. All data are summarized in Table 2.

Mesopancreatic (MP) infiltration was observed in 20 of 55 patients (36.4%) with ampullary carcinoma. It was significantly associated with venous invasion, which was present in 7 of 20 MP-positive patients (35.0%) and absent in all MP-negative cases (*p* < 0.001).

A similar association was observed with nodal involvement, detected in 15 of 20 MP-positive patients (75.0%) compared with 17 of 35 MP-negative patients (48.6%; *p* = 0.050).

Moreover, R0CRM− resection was achieved in 15 of 20 MP-positive and 34 of 35 MP-negative patients (75.0% vs. 97.1%; *p* = 0.020).

Other clinicopathological parameters demonstrated no statistically significant correlations with MP infiltration, although pancreatic parenchymal invasion exhibited a trend towards association (*p* = 0.091).

### 3.3. Mesopancreas and Distal Cholangiocarcinomas

Demographic data were correlated with the status of mesopancreatic infiltration in dCCA patients as well (Table 3).

Mesopancreatic (MP) infiltration was present in 28 of 45 patients (62.2%) with distal cholangiocarcinoma.

Cases with MP involvement showed a higher frequency of nodal metastases, observed in 20 patients (71.4%) compared with 4 patients (23.5%) without MP infiltration (*p* = 0.002).

Lymphatic vessel invasion occurred more often in MP-positive than in MP-negative tumours (17 patients, 60.7% vs. 4 patients, 23.5%; *p* = 0.030). Invasion of the pancreatic parenchyma also correlated closely with MP status, being detected in 26 patients (92.9%) with and in 10 patients (58.8%) without mesopancreatic involvement (*p* = 0.009). Regarding margin status, R0CRM− resection was achieved in 17 MP-positive cases (61.3%) and 15 MP-negative cases (88.2%), indicating a statistical trend (*p* = 0.088).

### 3.4. Correlation Analysis Between Topographic Tumour Infiltration Status and Tumour Entity (AC vs. dCCA)

The infiltration of the mesopancreas, the pancreatic parenchyma, and the duodenal wall was analyzed to compare the topographic tumour extension patterns between ampullary carcinoma (AC) and distal cholangiocarcinoma (dCCA). The results are summarized in Supplemental Table S1.

Mesopancreatic infiltration was significantly more frequent in dCCA than in AC patients (62.2% vs. 36.4%; *p* = 0.015).

A similar pattern was observed for pancreatic parenchymal invasion, detected in 36 of 45 dCCA patients (80.0%) compared with 24 of 55 AC patients (43.6%; *p* < 0.001).

In contrast, infiltration of the duodenal wall occurred more often in AC than in dCCA, with rates of 80.0% and 44.4%, respectively (*p* < 0.001).

### 3.5. Overall Survival in Ampullary Carcinoma (AC)

Overall survival was examined in relation to major clinicopathological variables (Table 4).

**Figure 2:**
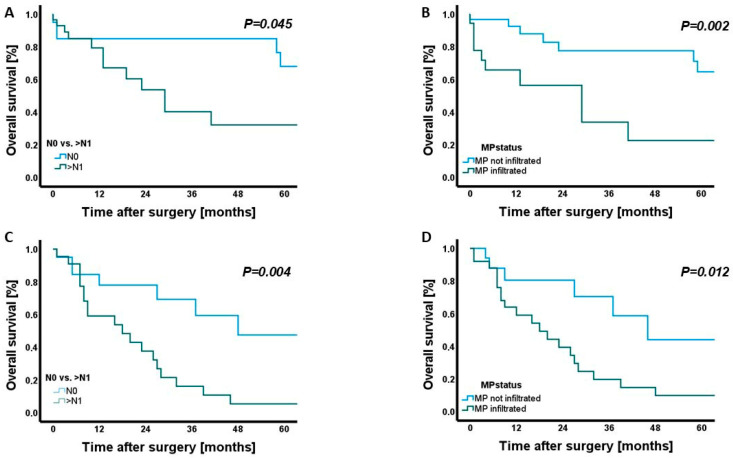
Kaplan–Meier analysis of overall survival stratified by lymph node status and mesopancreatic (MP) infiltration in ampullary carcinoma and distal cholangiocarcinoma (dCCA). (**A**) Overall survival in patients with ampullary carcinoma, comparing node-negative (N0) versus node-positive (>N1) disease (*p* = 0.045). (**B**) Overall survival in ampullary carcinoma stratified by mesopancreatic infiltration (MP not infiltrated vs. MP infiltrated; *p* = 0.002). (**C**) Overall survival in patients with distal cholangiocarcinoma (dCCA) according to lymph node status (N0 vs. >N1; *p* = 0.004). (**D**) Overall survival in dCCA based on mesopancreatic infiltration (MP not infiltrated vs. MP infiltrated; *p* = 0.012).

Both nodal involvement and mesopancreatic (MP) infiltration were associated with unfavourable survival outcomes (Figure 2A,B).

Patients with lymph node metastases demonstrated significantly shorter overall survival than those without nodal disease (*p* = 0.045). Similarly, MP infiltration was linked to a marked reduction in survival (*p* = 0.002). In multivariate Cox regression analysis, MP infiltration remained an independent predictor of poor overall survival (HR = 4.28, 95% CI 1.57–11.67, *p* = 0.005). Notably, the resection margin status (CRM0 vs. CRM+) did not correlate with overall survival (*p* = 0.592). No further parameters—including T category, histological grade, lymphatic, venous, or perineural invasion—showed a measurable association with overall survival.

### 3.6. Overall Survival in Distal Cholangiocarcinoma (dCCA)

In patients with distal cholangiocarcinoma, survival analysis revealed distinct prognostic effects for both nodal and mesopancreatic status (Table 5).

Overall survival was significantly shorter in node-positive compared with node-negative patients (*p* = 0.004), and likewise in MP-positive compared with MP-negative tumours (*p* = 0.012) (Figure 2C,D).

In the bivariate Cox regression model, nodal involvement emerged as the sole independent prognostic factor for overall survival (HR = 3.35, 95% CI 1.38–8.15, *p* = 0.008), whereas MP infiltration did not retain statistical significance when adjusted for nodal status. Comparable to AC, the resection margin status did not significantly influence overall survival.

## 4. Discussion

Periampullary carcinomas, comprising ampullary carcinoma (AC) and distal cholangiocarcinoma (dCCA), continue to exhibit limited long-term survival despite multimodal treatment [5,6,7]. Complete tumour clearance remains crucial, yet margin negativity is still difficult to achieve due to the complex anatomy of the pancreatic head region and the absence of established, compartment-oriented oncological standards [8,9,10,11,12,13]. As demonstrated in rectal and colon cancer, anatomically defined planes such as TME and CME can improve radicality and long-term outcomes [14,15,16].

Against this background, the mesopancreas (MP), an embryological compartment bordered by the Treitz and Fredet fasciae, has been proposed as such a compartment. Although the oncological relevance of the MP and the indication for mesopancreatic excision (MPE) remain a matter of debate, several studies suggest that the MP frequently serves as a central route of tumour extension in pancreatic ductal adenocarcinoma (PDAC), with infiltration rates of up to 80% [1,2,17,18]. Standardized MPE has been associated with improved R0 rates, increased lymph-node yield and reduced locoregional recurrence in PDAC patients [1,2,19], while meta-analytic data indicate no relevant increase in perioperative morbidity compared with conventional pancreatoduodenectomy [19]. The extent to which these concepts apply to periampullary carcinomas has so far remained undefined.

The aim of this study was to evaluate the infiltrative behaviour of AC and dCCA into the mesopancreas in order to investigate its histopathological and potential clinical relevance in these tumour entities, and to explore whether these findings may provide a first indication for the potential role of mesopancreatic excision. To our knowledge, there are no studies which have investigated the infiltrative behaviour of dCCAs and ACs into the mesopancreas.

This study was able to show that the mesopancreas is also infiltrated in patients with dCCAs (62.2%) and ACs (36.4%). While the MP infiltration rate is comparatively low in AC, we were able to demonstrate that the MP was infiltrated in dCCAs at a similar rate compared to PDAC patients (62.2% vs. 78.4%) [2]. In our opinion, this highlights the importance of considering the mesopancreas during surgery even for periampullary malignancies. Moreover, MP infiltration correlated with several adverse histopathological features: in AC, with nodal positivity, venous invasion, and higher margin positivity, and in dCCA with nodal metastasis, lymphatic invasion, and pancreatic parenchymal infiltration.

Notably, MP infiltration was significantly associated with incomplete resection status in ampullary carcinoma, whereas this association was not observed in distal cholangiocarcinoma. This discrepancy may be explained by fundamental differences in tumour growth patterns between these entities. Ampullary carcinomas may exhibit a contiguous mode of local invasion into adjacent structures [20], including the MP, such that MP involvement may directly reflect advanced local tumour extension and contribute to margin positivity. In contrast, distal cholangiocarcinomas more frequently demonstrate longitudinal spread along the bile duct, which may lead to margin involvement independent of MP infiltration [21]. Nevertheless, these findings do not preclude a potential role of mesopancreatic excision, but rather suggest that margin status in distal cholangiocarcinoma is determined by a more complex and multifactorial pattern of tumour spread.

Importantly, MP involvement was also associated with significantly reduced survival in both tumour entities. MP infiltration remained an independent prognostic factor in ampullary carcinoma, whereas this was not observed in distal cholangiocarcinoma after adjustment for nodal status. This difference may, in part, be explained by variations in clinical presentation and timing of diagnosis. Ampullary carcinomas are often detected at an earlier stage due to the early onset of symptoms, leading to earlier therapeutic intervention and a lower overall rate of mesopancreatic involvement. Consequently, the presence of MP infiltration in these patients may reflect a more advanced stage of disease and thus be associated with a poorer prognosis.

However, these findings must be interpreted with caution, as the relatively small cohort size, particularly after subgroup stratification, limits the robustness of multivariate analyses. Accordingly, both the absence of statistical significance in distal cholangiocarcinoma and the observed significance in ampullary carcinoma may be influenced by limited statistical power.

Together, these findings indicate that MP infiltration may reflect biologically aggressive disease and support considering the mesopancreas as a relevant parameter in prognostic assessment and preoperative evaluation. Previous studies, including contributions from our group and others, have demonstrated in pancreatic ductal adenocarcinoma that distinct radiological features on preoperative imaging, such as mesopancreatic fat stranding and increased tissue density, may be associated with histopathologically confirmed mesopancreatic involvement [22,23,24]. While these findings suggest that imaging-based assessment of the mesopancreas may be feasible, their applicability to periampullary malignancies remains to be established. In line with these considerations, the mesopancreas has increasingly gained attention as a potential anatomical compartment of interest in surgical oncology.

In the current literature, MPE has been compared with conventional pancreatoduodenectomy without determining the actual mesopancreatic infiltration status as a stratification parameter. Two studies by Quero et al., one including periampullary carcinomas and another focusing specifically on ampullary carcinoma, consistently demonstrated improved oncological radicality with MPE, reflected by a higher lymph-node yield and substantially lower local recurrence rates. Both studies demonstrated only trends to improved DFS and OS; neither reported significant differences in operative time, blood loss or postoperative morbidity, underscoring the safety of the technique [25,26]. These findings are consistent with our results, which likewise show that mesopancreatic involvement is associated with significantly worse survival in both AC and dCCA. Moreover, our study adds new information by directly analyzing mesopancreatic infiltration and by characterizing an important pattern of tumour spread, which complements the previously reported benefits of mesopancreatic excision. Kawabata et al. [29] retrospectively investigated in 74 patients whether MPE provides an oncological benefit in patients with dCCAs and revealed similar results [29]. However, in these studies, the infiltration status of the MP remained unknown.

Compared to the known literature for the resection status after oncological surgery in patients with dCCAs, there was no superior difference compared to our results [9,10,11,13,29,30,31,32]. In particular, a meta-analysis by Zhou et al., who investigated the resection rates after surgery for dCCA, showed a R0 rate of 84%, with 39 incorporated studies comprising 3258 patients in total. However, it was not mentioned whether a systematic CRM evaluation was implemented or not [13]. The lack of a significant association between resection margin status and overall survival in our cohort contrasts with parts of the existing literature [13,33]. One possible explanation is the strict application of the CRM-based definition using the 1 mm rule, which increases the proportion of R1 resections and may reduce the discriminatory power between R0 and R1 groups. In addition, the relatively small cohort size, together with the strong influence of tumour biology, particularly nodal involvement and other markers of advanced disease, may have limited statistical power and reduced the ability to discriminate survival differences between margin-defined groups. Finally, the biological heterogeneity of periampullary tumours and potential differences in adjuvant treatment strategies may further contribute to the inconsistent prognostic relevance of margin status observed in this cohort.

Considering only ampullary carcinomas, propagated R0 resection rates are even higher in the literature. Most patients with ACs show symptoms in an early stage of disease, resulting from the proximity of the AC to the sphincter Oddi [33]. Our data underlines this hypothesis. Only 36.4% of all AC patients showed an infiltration of the mesopancreas, which was significantly lower compared to both dCCA and PDAC patients [2].

Some data in the literature propagate that pancreatoduodenectomy is not necessary for all stages of ampullary carcinomas. However, a meta-analysis by Heise et al., who investigated the resection rates after interventional or surgical therapy in 3829 patients with ampullary cancer, showed that pancreaticoduodenectomy most reliably achieved margin clearance in ampullary carcinoma, with Ref. [32] underlining that these tumours commonly extend outwards the periampullary region. In our cohort of 55 AC patients, the pancreas and the duodenum were infiltrated in 43.6% and 80.0%, respectively. In our opinion, radical surgery is inevitable unless preoperative staging definitely predicts that a less radical approach will achieve complete oncological resection. With a significant low rate of mesopancreatic infiltration, MPE remains a controversial approach for AC patients. However, it remains unknown how to stratify these patients preoperatively and rule out mesopancreatic infiltration.

This study has several limitations that should be acknowledged. First, its retrospective, single-centre design may limit the generalisability of the findings. Second, the overall sample size was relatively small, particularly after stratification into tumour subgroups and survival analyses. This is largely attributable to the low incidence of periampullary carcinomas, but it nevertheless restricts the statistical power of the study and the strength of the conclusions. Post-recurrence management was based on interdisciplinary tumour board recommendations; however, implementation and adherence were heterogeneous, which may have influenced overall survival outcomes. Therefore, our findings should be interpreted as exploratory and hypothesis-generating. Larger, ideally multicentre studies are needed to validate the clinical and prognostic relevance of mesopancreatic infiltration in ampullary carcinoma and distal cholangiocarcinoma. In this study, the status of mesopancreatic infiltration was evaluated independent on the location (ventral, dorsal, dorsomedial) and depth of invasion. The aim of this study was to reveal the primary incidental significance of the infiltration magnitude towards the mesopancreatic area in periampullary identities, which was previously unknown. Taking our results together, the high MP infiltration rate suggests that MPE is useful not only for PDAC, but for periampullary carcinomas as well especially in dCCAs. Further studies are needed to evaluate the exact extent and location of invasion. Since we presume that primary resectable PDAC patients remain at risk for mesopancreatic infiltration and that the mesopancreas could serve as a further stratification marker, it seems necessary to investigate this further for periampullary cancers as well.

## 5. Conclusions

This is the first study to histopathologically characterize mesopancreatic infiltration in periampullary carcinomas. Mesopancreatic involvement is frequent in distal cholangiocarcinoma and, although less common, also present in ampullary carcinoma, and is consistently associated with adverse histopathological features and significantly reduced overall survival. These findings suggest that the mesopancreas may represent a relevant oncological compartment in periampullary malignancies. However, the implications for surgical strategy, including mesopancreatic excision, remain investigational and require validation in prospective, entity-specific studies. In future, mesopancreatic status could evolve into a perioperative staging variable to guide individualized surgical strategies and the extent of resection.

## Figures and Tables

**Table 1:** Demographic data of all 100 patients. Staging is revised to the 8th edition of the UICC TNM classification of malignant tumours. Statistical significance was calculated using the Fisher Exact Test (*p* ≤ 0.05 is significant). ** indicates a *p*-value ≤ 0.01; * indicates a *p*-value ≤ 0.05.

	dCCA n = 45		AC n = 55		*p*-Value
**Age in years** (median (range))	68 (40–84)		66 (31–83)		
**Sex**	**n**	**%**	**n**	**%**	*0.018* *
Female	9	20.0	24	43.6	
Male	36	80.0	31	56.4	
**T-Status**					*0.003* *
T1(a/b)	3	6.7	16	29.1	
T2	9	20.0	17	30.9	
T3(a/b)	31	68.9	20	36.4	
T4	2	4.4	2	3.6	
**N-Status**					*0.696*
N0	21	46.7	23	41.8	
N1	15	33.3	23	41.8	
N2	9	20.0	9	16.4	
**G-Status**					*0.971*
G1	3	6.7	3	5.5	
G2	25	55.6	30	54.5	
G3	17	37.7	21	38.2	
unknown	0	0.0	1	1.8	
**Pn-Status**					*<0.001* **
Pn0	15	33.3	40	72.7	
Pn1	30	66.7	15	27.3	
**V-Status**					*0.577*
V0	37	82.2	48	87.3	
V1	8	17.8	7	12.7	
**L-Status**					*0.999*
L0	24	53.3	30	54.5	
L1	21	46.7	25	45.5	
**R-status**					*0.039* *
R0CRM−	32	71.1	49	89.1	
R1/R0CRM+	13	28.9	6	10.9	

Pn: perineural invasion; L: lymphatic invasion; V: venous invasion.

**Table 2:** Correlation analysis of the 55 AC patients stratified according to infiltration status into the mesopancreas (MP). Statistical significance was calculated by the chi squared test and Wilcoxon test. ** indicates a *p*-value ≤ 0.01; * indicates a *p*-value ≤ 0.05.

	MP Infiltration + n = 20		MP Infiltration − n = 35		*p*-Value
**Sex**	**n**	**%**	**n**	**%**	*0.403*
Female	7	35.0	17	48.6	
Male	13	65.0	18	51.4	
**T-Status**					*0.163*
≤T2	9	45.0	23	65.7	
≥T3	11	55.0	12	32.3	
**N-Status**					*0.050* *
N0	5	25.0	18	51.4	
N1/N2	15	75.0	17	48.6	
**G-Status**					*0.566*
G1/2	10	50.0	22	62.9	
G3	9	45.0	13	37.1	
unknown	1	5.0	-	-	
**Pn-Status**					*0.128*
Pn0	12	60.0	28	80.0	
Pn1	8	40.0	7	20.0	
**V-Status**					*<0.001* **
V0	13	65.0	35	100.0	
V1	7	35.0	0	0	
**L-Status**					*0.159*
L0	8	40.0	22	62.9	
L1	12	60.0	13	37.1	
**Infiltrated PP**					*0.091*
positive	12	60.0	12	32.3	
negative	8	40.0	23	65.7	
**Infiltrated Duo**					*0.728*
Positive	17	85.0	27	77.1	
negative	3	15.0	8	22.9	
**Resection Margin**					*0.020* *
R0CRM−	15	75.0	34	97.1	
R0CRM+/R1	5	25.0	1	2.9	

Duo: duodenum; L: lymphatic invasion; MP: mesopancreas; Pn: perineural invasion; V: venous invasion; PP: pancreas parenchyma.

**Table 3:** Correlation analysis of the 45 dCCA patients stratified according to infiltration status into the mesopancreas (MP). Statistical significance was calculated by the chi squared test and Wilcoxon test. ** indicates a *p*-value ≤ 0.01; * indicates a *p*-value ≤ 0.05.

	MP Infiltration + n = 28		MP Infiltration − n = 17		*p*-Value
**Sex**	**n**	**%**	**n**	**%**	*0.447*
Female	7	25.0	2	11.8	
Male	21	75.0	15	88.2	
**T-Status**					*0.072*
≤T2	4	14.3	7	41.2	
≥T3	24	85.7	10	58.8	
**N-Status**					*0.002* **
N0	8	28.6	13	76.5	
N1/N2	20	71.4	4	23.5	
**G-Status**					*0.118*
G1/2	14	50.0	13	76.5	
G3	14	50.0	4	23.5	
**Pn-Status**					*0.193*
Pn0	7	25.0	8	47.1	
Pn1	21	75.0	9	52.9	
**V-Status**					*0.132*
V0	21	75.0	16	94.1	
V1	7	25.0	1	5.9	
**L-Status**					*0.030* *
L0	11	39.3	13	76.5	
L1	17	60.7	4	23.5	
**Infiltrated PP**					*0.009* **
positive	26	92.9	10	58.8	
negative	2	7.1	7	41.2	
**Infiltrated Duo**					*0.372*
Positive	14	50.0	6	35.3	
negative	14	50.0	11	64.7	
**Resection Margin**					*0.088*
R0CRM−	17	61.3	15	88.2	
R0CRM+/R1	11	39.3	2	11.8	

Duo: duodenum; L: lymphatic invasion; MP: mesopancreas; Pn: perineural invasion; V: venous invasion; PP: pancreas parenchyma.

**Table 4:** Univariate and multivariate analysis of individual clinical and histopathological parameters for AC regarding overall survival.

Univariate analysis			
		*p*-value	
Sex (male vs. female)		*0.104*	
T-stage (T1/T2 vs. T3/T4)		*0.958*	
N-stage (N0 vs. >N0)		*0.045*	
Grading (G1 vs. G2/G3)		*0.751*	
Pn (Pn0 vs. Pn1)		*0.986*	
L (L0 vs. L1)		*0.915*	
V (V0 vs. V1)		*0.009*	
R-status (R0CRM− vs. R0CRM+)		*0.592*	
MP-status (MP+ vs. MP−)		*0.002*	
Multivariate analysis			
	*p*-value	HR	95% CI
MP-status (MP+ vs. MP−)	*0.005*	4.28	1.57–11.67

CRM = circumferential resection margin, MP-status = mesopancreatic infiltration status.

**Table 5:** Univariate and bivariate analysis of individual clinical and histopathological parameters for dCCA regarding overall survival.

Univariate analysis			
		*p*-value	
Sex (male vs. female)		*0.794*	
T-stage (T1/T2 vs. T3/T4)		*0.958*	
N-stage (N0 vs. N1)		*0.004*	
Grading (G1 vs. G2/G3)		*0.219*	
Pn (Pn0 vs. Pn1)		*0.758*	
L (L0 vs. L1)		*0.151*	
V (V0 vs. V1)		*0.978*	
R-status (R0CRM− vs. R0CRM+)		*0.291*	
MP-status (MP+ vs. MP−)		*0.012*	
Bivariate analysis			
	*p*-value	HR	95% CI
N-status (N0 vs. >N0)	*0.008*	3.35	1.38–8.15

CRM = circumferential resection margin, MP-status = mesopancreatic infiltration status.

## Data Availability

The data are not publicly available due to privacy and ethical restrictions but are available from the corresponding author on reasonable request.

## Supplementary Materials

The following supporting information can be downloaded at: https://www.mdpi.com/article/10.3390/cancers18091434/s1, Table S1: Correlation analysis between tumour infiltration status (MP, PP and Duo) and tumour entity (AC vs. dCCA).

## Abbreviations

The following abbreviations are used in this manuscript:

AC	Ampullary carcinoma
CRM	Circumferential resection margin
dCCA	Distal cholangiocarcinoma
Duo	Duodenum
hPDAC	Pancreatic head ductal adenocarcinoma
L	Lymphatic invasion
LEEPP	Leeds Pathology Protocol
MP	Mesopancreatic
MPE	Mesopancreatic excision
PD	Pancreatoduodenectomy
Pn	Perineural invasion
PP	Pancreatic parenchyma
V	Venous invasion

## References

[1] Safi S.A., David S., Haeberle L., Vaghiri S., Luedde T., Roderburg C., Esposito I., Fluegen G., Knoefel W.T. (2025). Most oncological pancreas resections must consider the mesopancreas. BMC Cancer.

[2] Safi S.A., Haeberle L., Fluegen G., Lehwald-Tywuschik N., Krieg A., Keitel V., Luedde T., Esposito I., Rehders A., Knoefel W. (2021). Mesopancreatic excision for pancreatic ductal adenocarcinoma improves local disease control and survival. Pancreatology.

[3] Garcia-Granero A., Pellino G., Frasson M., Fletcher-Sanfeliu D., Bonilla F., Sánchez-Guillén L., Dolz A.D., Romaguera V.P., Ortí L.S., Martinez-Soriano F. (2019). The fusion fascia of Fredet: An important embryological landmark for complete mesocolic excision and D3-lymphadenectomy in right colon cancer. Surg. Endosc..

[4] Cong L., Liu Q., Zhang R., Cui M., Zhang X., Gao X., Guo J., Dai M., Zhang T., Liao Q. (2018). Tumor size classification of the 8(th) edition of TNM staging system is superior to that of the 7(th) edition in predicting the survival outcome of pancreatic cancer patients after radical resection and adjuvant chemotherapy. Sci. Rep..

[5] Esnaola N.F., Meyer J.E., Karachristos A., Maranki J.L., Camp E.R., Denlinger C.S. (2016). Evaluation and management of intrahepatic and extrahepatic cholangiocarcinoma. Cancer.

[6] Lee R.M., Maithel S.K. (2019). Approaches and Outcomes to Distal Cholangiocarcinoma. Surg. Oncol. Clin. N. Am..

[7] Umino R., Nara S., Mizui T., Takamoto T., Ban D., Esaki M., Hiraoka N., Shimada K. (2024). ASO Author Reflections: Future Perspectives in Surgical Management of Distal Cholangiocarcinoma: Insights from Surgical Margin Status and Recurrence Patterns. Ann. Surg. Oncol..

[8] Chen Z., Yu B., Bai J., Li Q., Xu B., Dong Z., Zhi X., Li T. (2021). The Impact of Intraoperative Frozen Section on Resection Margin Status and Survival of Patients Underwent Pancreatoduodenectomy for Distal Cholangiocarcinoma. Front. Oncol..

[9] Hatzaras I., George N., Muscarella P., Melvin W.S., Ellison E.C., Bloomston M. (2010). Predictors of survival in periampullary cancers following pancreaticoduodenectomy. Ann. Surg. Oncol..

[10] Menon K.V., Gomez D., Smith A.M., Anthoney A., Verbeke C.S. (2009). Impact of margin status on survival following pancreatoduodenectomy for cancer: The Leeds Pathology Protocol (LEEPP). HPB.

[11] van Roest M.H., Gouw A.S., Peeters P.M., Porte R.J., Slooff M.J., Fidler V., De Jong K.P. (2008). Results of pancreaticoduodenectomy in patients with periampullary adenocarcinoma: Perineural growth more important prognostic factor than tumor localization. Ann. Surg..

[12] Westgaard A., Tafjord S., Farstad I.N., Cvancarova M., Eide T.J., Mathisen O., Clausen O.P.F., Gladhaug I.P. (2008). Pancreatobiliary versus intestinal histologic type of differentiation is an independent prognostic factor in resected periampullary adenocarcinoma. BMC Cancer.

[13] Zhou Y., Liu S., Wu L., Wan T. (2017). Survival after surgical resection of distal cholangiocarcinoma: A systematic review and meta-analysis of prognostic factors. Asian J. Surg..

[14] Hohenberger W., Weber K., Matzel K., Papadopoulos T., Merkel S. (2009). Standardized surgery for colonic cancer: Complete mesocolic excision and central ligation–technical notes and outcome. Color. Dis..

[15] West N.P., Anderin C., Smith K.J., Holm T., Quirke P. (2010). Multicentre experience with extralevator abdominoperineal excision for low rectal cancer. Br. J. Surg..

[16] Bertelsen C.A., Bols B., Ingeholm P., Jansen J.E., Neuenschwander A.U., Vilandt J. (2011). Can the quality of colonic surgery be improved by standardization of surgical technique with complete mesocolic excision?. Color. Dis..

[17] Safi S.A., Alexander A., Neuhuber W., Haeberle L., Rehders A., Luedde T., Esposito I., Fluegen G., Knoefel W.T. (2024). Defining distal splenopancreatectomy by the mesopancreas. Langenbeck’s Arch. Surg..

[18] Kawabata Y., Tanaka T., Nishi T., Monma H., Yano S., Tajima Y. (2012). Appraisal of a total meso-pancreatoduodenum excision with pancreaticoduodenectomy for pancreatic head carcinoma. Eur. J. Surg. Oncol. (EJSO).

[19] da Silva L.F.L., Belotto M., de Almeida L.F.C., Samuel J., Pereira L.H., Albagli R.O., de Araujo M.S., Ramia J.M. (2024). Radicality and safety of total mesopancreatic excision in pancreatoduodenectomy: A systematic review and meta-analysis. World J. Surg. Oncol..

[20] Miura Y., Ohgi K., Ohike N., Ashida R., Yamada M., Otsuka S., Kato Y., Norose T., Sugino T., Uesaka K. (2024). Clinical Implications of the Degree of Pancreatic Invasion in Ampulla of Vater Carcinoma. Ann. Surg. Oncol..

[21] Guedj N. (2022). Pathology of Cholangiocarcinomas. Curr. Oncol..

[22] Safi S.A., Haeberle L., Heuveldop S., Kroepil P., Fung S., Rehders A., Keitel V., Luedde T., Fuerst G., Esposito I. (2021). Pre-Operative MDCT Staging Predicts Mesopancreatic Fat Infiltration-A Novel Marker for Neoadjuvant Treatment?. Cancers.

[23] David S., Safi S., Fink B., Pustu I., Vaghiri S., Sultani A., Alexander A., Wolf-Vollenbroeker M., Haeberle-Graser L., Roderburg C. (2026). Standardizing computed tomographic assessment of the mesopancreas in pancreatic cancer patients. Abdom. Radiol..

[24] Wellner U.F., Krauss T., Csanadi A., Lapshyn H., Bolm L., Timme S., Kulemann B., Hoeppner J., Kuesters S., Seifert G. (2016). Mesopancreatic Stromal Clearance Defines Curative Resection of Pancreatic Head Cancer and Can Be Predicted Preoperatively by Radiologic Parameters: A Retrospective Study. Medicine.

[25] Quero G., Fiorillo C., De Sio D., Laterza V., Menghi R., Cina C., Schena C.A., Rosa F., Galiandro F., Alfieri S. (2021). The role of mesopancreas excision for ampullary carcinomas: A single center propensity-score matched analysis. HPB.

[26] Quero G., Fiorillo C., Menghi R., Cina C., Galiandro F., Longo F., Sofo F., Rosa F., Tortorelli A.P., Giustiniani M.C. (2020). Total mesopancreas excision for periampullary malignancy: A single-center propensity score-matched comparison of long-term outcomes. Langenbeck’s Arch. Surg..

[27] Kimura W. (2000). Surgical anatomy of the pancreas for limited resection. J. Hepato-Biliary-Pancreat. Surg..

[28] Gockel I., Domeyer M., Wolloscheck T., Konerding M.A., Junginger T. (2007). Resection of the mesopancreas (RMP): A new surgical classification of a known anatomical space. World J. Surg. Oncol..

[29] Kawabata Y., Hayashi H., Ishikawa N., Tajima Y. (2016). Total meso-pancreatoduodenum excision with pancreaticoduodenectomy in lower biliary tract cancer. Langenbeck’s Arch. Surg..

[30] Verbeke C.S., Leitch D., Menon K.V., McMahon M.J., Guillou P.J., Anthoney A. (2006). Redefining the R1 resection in pancreatic cancer. Br. J. Surg..

[31] Jarufe N.P., Coldham C., Mayer A.D., Mirza D.F., Buckels J.A., Bramhall S.R. (2004). Favourable prognostic factors in a large UK experience of adenocarcinoma of the head of the pancreas and periampullary region. Dig. Surg..

[32] Heise C., Abou Ali E., Hasenclever D., Auriemma F., Gulla A., Regner S., Gaujoux S., Hollenbach M. (2020). Systematic Review with Meta-Analysis: Endoscopic and Surgical Resection for Ampullary Lesions. J. Clin. Med..

[33] Nießen A., Loos M., Neumüller K., Feißt M., Klaiber U., Cizmic A., Al-Saeedi M., Roth S., Schneider M., Büchler M.W. (2023). Impact of circumferential resection margin on survival in ampullary cancer: Retrospective analysis. BJS Open.

