# Supplementary material for: The Role of the Mesopancreas in Periampullary Malignancies
Source: Cancers (Basel). 2026 Apr 30;18(9):1434. doi: 10.3390/cancers18091434 (PMC13162973; doi:10.3390/cancers18091434)
Supplement: Supplementary file 1 [file cancers-18-01434-s001.zip › cancers-4237290-supplementary.pdf]

cancers

MDPI

Supplemental Materials

The Role of the Mesopancreas in Periampullary Malignancies

Table S1. Correlation analysis between tumor infiltration status (MP, PP and Duo) and tumor entity (AC vs. dCCA). Statistical significance calculated using the Fisher Exact Test. (** indicates a *p-value ≤ 0.01*; * indicates a *p-value ≤ 0.05)*.

	**AC n = 55**		**dCCA n = 45**		
	**MP Infiltration**				***p-value***
	n	%	n	%	
**Infiltration positive** ion	20	36.4	28	62.2	***0.015****
**Infiltration negative**	35	63.6	17	37.8	
	**PP Infiltration**				
**Infiltration positive** tion	24	43.6	36	80.0	***< 0.001*****
**Infiltration negative** keine Infiltration	31	56.4	9	20.0	
	**Duo Infiltration**				
**Infiltration positive** ion	44	80.0	20	44.4	***< 0.001*****
**Infiltration negative**	11	20.0	25	55.6	
